# Rare Case of Paradoxical Embolism Through Anomalous Systemic Vein-to-Pulmonary Vein Connection: When Zebras Are Zebras

**DOI:** 10.1016/j.jaccas.2025.104010

**Published:** 2025-07-16

**Authors:** Jacob Y. Cao, Nathan K.P. Wong, Phillip Lo, Stephanie Wiltshire, Nicholas Presgrave, Mayooran Namasivayam, Henora Chour, David Boshell, Stephen Tisch, David Roy

**Affiliations:** a Department of Cardiology, St Vincent’s Hospital, Sydney, Australia; b School of Medicine, University of New South Wales, Sydney, Australia; c Department of Neurology, St Vincent’s Hospital, Sydney, Australia; d Department of Radiology, St Vincent’s Hospital, Sydney, Australia

**Keywords:** bubble study, paradoxical embolism, systemic vein-to-pulmonary vein connection, transcatheter intervention

## Abstract

We report a unique case of a congenital anomalous systemic vein-to-pulmonary vein connection with bidirectional flow causing paradoxical embolic stroke. A 64-year-old woman with no significant medical history presented with a multiterritorial embolic stroke after ankle fracture surgery. Computed tomography (CT) revealed occlusion of the left middle cerebral artery (MCA) and ischemia in the right MCA territory. Successful endovascular clot retrieval was performed, targeting the left MCA occlusion. Extensive investigation identified a rare connection between the right lower pulmonary vein and suprahepatic inferior vena cava, confirmed through transesophageal echocardiography, CT pulmonary angiogram, and invasive angiography. This connection facilitated right-to-left shunting, leading to paradoxical embolism. The connection was closed successfully with an Amplatzer vascular plug, eliminating the shunt. This case underscores the importance of considering rare anatomical anomalies in cryptogenic stroke and demonstrates the feasibility of device closure for managing such connections to prevent recurrent embolic events.

## Graphical Abstract

**Figure:**
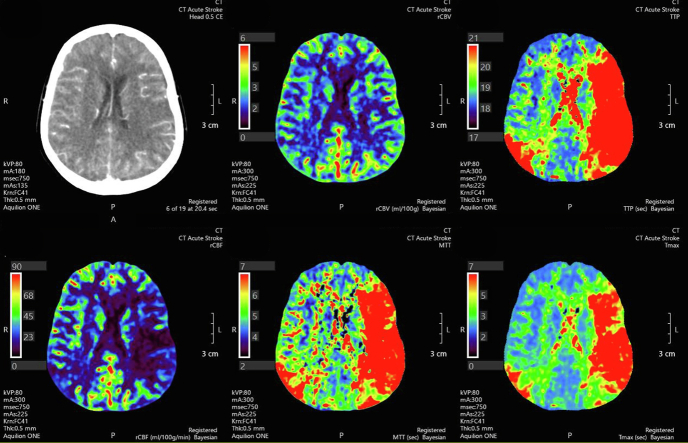

## History of Presentation

**Figure 1:**
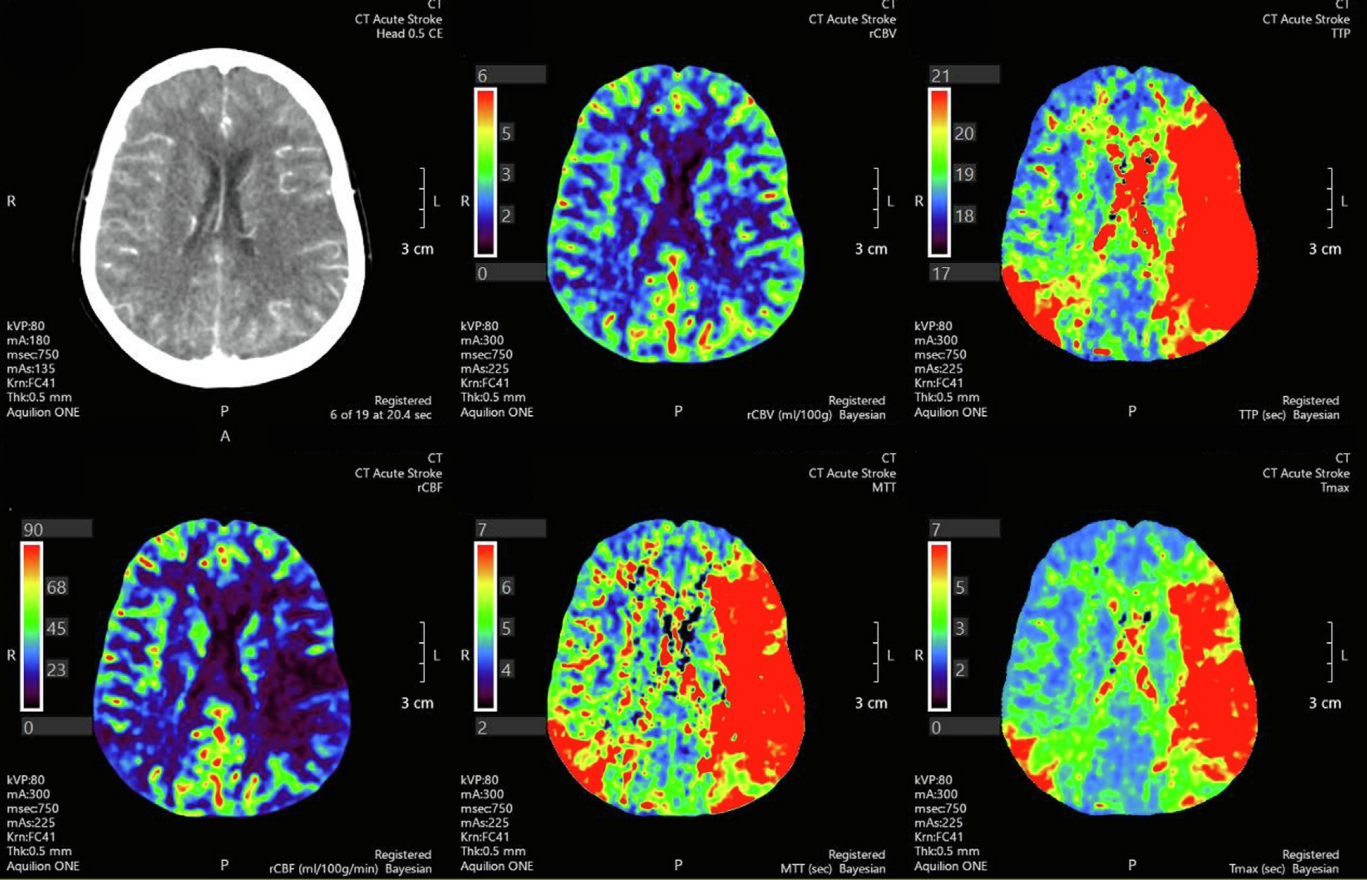
Brain Computed Tomography Computed tomography showing a large multiterritorial ischemic stroke.

A 64-year-old woman presented with a traumatic ankle fracture. Initial management involved immobilization and external fixation, with low-molecular-weight heparin for deep vein thrombosis prophylaxis. Six days after presentation, she underwent uneventful internal fixation. Two hours postoperatively, she developed new expressive aphasia and right facial droop. Urgent brain computed tomography (CT) demonstrated an acute left middle cerebral artery (M1) occlusion, with large ischemic penumbra and minimal infarct core (Figure 1). There were additional changes of reduced perfusion affecting the right middle cerebral arterial territory but without large-vessel occlusion. Successful endovascular clot retrieval was performed with a significant improvement in expressive dysphasia. A thorough evaluation was initiated to determine the etiology of the stroke.

**Take-Home Messages**

• Multiterritorial strokes without a clear source should prompt evaluation for rare right-to-left shunting mechanisms, including systemic vein-to-pulmonary vein anomalies.
• Transesophageal echocardiography with a bubble study through a lower limb cannula, combined with cross-sectional imaging, is essential for diagnosing elusive causes of paradoxical embolism.

## Medical History

The patient had diet-managed hypercholesterolemia but no other significant medical history. She had 3 previous pregnancies with no complications. She was not taking any regular medications.

## Differential Diagnosis

The multiterritorial nature of the stroke raised suspicion for a cardioembolic source. Initial considerations included atrial fibrillation, patent foramen ovale, or other right-to-left shunts, either intra- or extracardiac. Embolic sources from the aortic arch or extracranial vessels were also believed to be unlikely given no significant atheroma noted on the CT angiograms.

## Investigations

Laboratory studies were unremarkable, with mildly abnormal lipid levels (total cholesterol 186 mg/dL, low-density-lipoprotein cholesterol 112 mg/dL, triglycerides 124 mg/dL, high-density-lipoprotein cholesterol 50 mg/dL). Her thrombophilia screen found that she was heterozygous for the Factor V Leiden mutation (G1691A), which is associated with elevated venous thromboembolic risk, especially in the context of another precipitating factor.^1^

Electrocardiogram demonstrated sinus rhythm with no evidence of atrial fibrillation on prolonged cardiac monitoring. Lower limb Doppler ultrasonography performed 5 days after endovascular clot retrieval showed no evidence of deep vein thrombosis, although visualization of the right leg was limited by the presence of a cast.

A transthoracic echocardiogram with a bubble study through an upper limb cannula was negative for right-to-left shunting under Valsalva. Given high clinical suspicion, transesophageal echocardiography was pursued, which demonstrated normal biventricular size and function, normal biatrial sizes, and normal valvular function. The foramen ovale was not hypermobile or aneurysmal, with no shunting noted on color Doppler (Video 1). A bubble study using agitated saline solution through a lower limb cannula was strongly positive, with bubbles visualized in the left atrium within 3 cardiac cycles. However, the bubbles did not appear to traverse the interatrial septum (Video 2).

**Figure 2:**
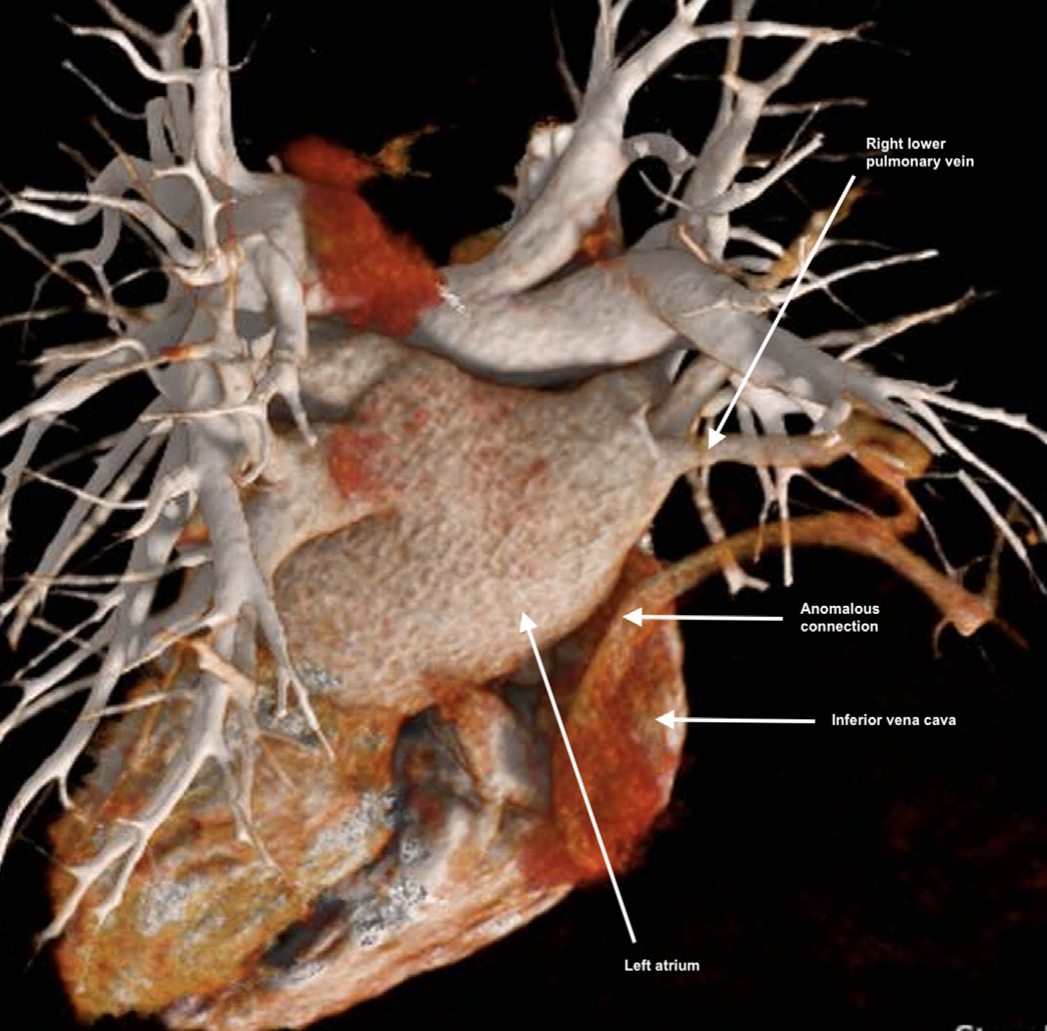
Anomalous Pulmonary Vein-Inferior Vena Cava Connection as Cause of Paradoxical Embolism Three-dimensional computed tomography reconstruction showing anomalous connection between the suprahepatic inferior vena cava and the right lower pulmonary vein.

Subsequent CT pulmonary angiogram revealed a possible anomalous connection between the right lower pulmonary vein and the inferior vena cava (IVC) (Figure 2, Video 3). Contrast was noted in the pulmonary arteries and veins, in addition to within this connection and the IVC, but not elsewhere in the systemic circulation, suggestive of left-to-right shunting at the time of CT. Together with the positive bubble study, we concluded that this anomalous connection allowed for bidirectional flow depending on the left and right atrial pressures, thereby permitting paradoxical embolism. No other extracardiac abnormalities were noted. Invasive angiography confirmed an anomalous vein arising from the suprahepatic IVC and draining into the right lower pulmonary vein (Video 4).

## Management

The anomalous venous connection was identified as the likely source of paradoxical embolism in the setting of suspected venous thrombosis precipitated by trauma and surgery. Closure of the anomalous connection was performed using an Amplatzer AVP1 (10 mm × 7 mm) vascular plug, achieving complete occlusion without residual leaks (Video 5).

Postprocedure, the patient continued speech rehabilitation and is expected to make an excellent recovery.

The patient will remain on therapeutic anticoagulation with apixaban for at least 3 to 6 months. A repeat CT pulmonary angiogram will be performed to confirm involution of the anomalous connection before anticoagulation is discontinued. The patient gave verbal and written consent to the sharing of anonymized information highlighted above.

## Discussion

This case represents, to our knowledge, the first reported instance of a congenital anomalous systemic vein-to-pulmonary vein connection causing a paradoxical embolic stroke. Such connections can be either congenital or acquired.

Acquired anomalous systemic vein-to-pulmonary vein connections are typically associated with conditions that result in obstructed systemic venous return (eg, superior vena cava thrombosis or extrinsic compression) or elevated central venous pressure (eg, Fontan circulation).^2^^,^^3^ These conditions lead to the development of physiological collaterals. However, our patient had no clinical evidence or history of these contributing factors.

Congenital anomalous connections can occur as part of partial anomalous pulmonary venous drainage, such as in Scimitar syndrome, which involves partial anomalous drainage of the right-sided pulmonary veins into the IVC.^4^ Scimitar syndrome is often associated with right lung hypoplasia and other cardiac anomalies. However, paradoxical embolism is not typically associated with isolated partial anomalous drainage of the pulmonary vein because the lungs act as a clot filter, preventing systemic embolism.

In this unique case, the patient had 4 pulmonary veins draining normally into the left atrium, but an anomalous connection existed between the right lower pulmonary vein and the suprahepatic IVC. This connection allowed for intermittent right-to-left shunting and paradoxical embolism, bypassing the pulmonary capillary filter.

Unique to this case was the bidirectional flow within this anomalous connection, evident through a combination of the CT pulmonary angiogram and bubble study. Acquired systemic vein-to-pulmonary vein collaterals typically shunt right-to-left because of elevated systemic venous pressures. Such shunts often present with hypoxia, left-sided volume overload, and, rarely, paradoxical embolism. In contrast, our patient had normal cardiac anatomy and function, with likely comparable pressures in the systemic venous and left atrial systems. This equilibrium limited shunting through the anomalous connection to an intermittent basis. The shunt produced no significant hemodynamic burden, which may explain the delayed clinical presentation as a thromboembolic event rather than a hemodynamic complication.

## Conclusions

This case underscores the importance of considering rare anatomical anomalies in the differential diagnosis of cryptogenic stroke, particularly when conventional sources of embolism are not identified. Furthermore, it highlights the utility of advanced imaging and invasive diagnostic techniques in uncovering elusive anomalies responsible for paradoxical embolism.

## Funding Support and Author Disclosures

The authors have reported that they have no relationships relevant to the contents of this paper to disclose.

## Appendix

Video 1 Transesophageal echocardiogram showing a normal-appearing interatrial septum with no shunting on color Doppler interrogation.

Video 2 Transesophageal echocardiogram showing a strongly positive bubble study through a lower limb cannula.

Video 3 Transverse computed tomography sections showing the anomalous connection between the suprahepatic inferior vena cava and right lower pulmonary vein.

Video 4 Invasive angiography with catheter inserted through the femoral vein and the catheter tip located in the anomalous connection. Contrast injection showed right-to-left shunting through this connection into the left heart chambers.

Video 5 Invasive angiography showing successful occlusion of the anomalous connection just distal to its take-off from the inferior vena cava.
